# Operations and Christian Science

**Published:** 1912-09-21

**Authors:** Frederick Dixon

**Affiliations:** District Office, Christian Science Committees on Publication. 23-29 Norfolk Street, Strand


### The Editor's Letter-Box.

### Operations and Christian Science.

*To the Editor of* The Hospital.

Sir,—In your issue of the 14th inst. there is a note on the subject of operations and Christian Science. I think most Christian Scientists will be likely to share your amazement on the subject. When a gentleman enters a hospital, undergoes an operation, takes medicine, and accepts all the attention which the organisation has to offer him, it would perhaps be as well if he ceased to talk about Christian Science.

I think you will agree with me that, if that had been typical of the Christian Science movement, it would not have assumed the proportions it has to-day. I am glad, however, that the gentleman expressed his gratitude for the aid he received. That seems to have been the one feature in the business in consonance with Christian Science.—Yours truly, Frederick Dixon,

District Office, Christian Science Committees on Publication.

23-29 Norfolk Street, Strand, September 14.

